# Experiment and Numerical Simulation on Gas-Liquid Annular Flow through a Cone Sensor

**DOI:** 10.3390/s18092923

**Published:** 2018-09-03

**Authors:** Denghui He, Senlin Chen, Bofeng Bai

**Affiliations:** 1 State Key Laboratory of Eco-Hydraulic in Northwest Arid Region, Xi’an University of Technology, Xi’an 710048, China; chensl@xaut.edu.cn; 2 State Key Laboratory of Multiphase Flow in Power Engineering, Xi’an Jiaotong University, Xi’an 710049, China

**Keywords:** gas-liquid annular flow, cone sensor, pressure recovery length, vortex, VOF coupled DPM

## Abstract

The cone meter has been paid increasing attention in wet gas measurement, due to its distinct advantages. However, the cone sensor, which is an essential primary element of the cone meter, plays a role in the measurement of wet gas flow that is important, but not fully understood. In this article, we investigate the gas-liquid annular flow through a cone sensor by experiment and numerical simulation. Emphasis is put on the influences of pressure recovery characteristics and flow structure, and how they are affected by the cone sensor. The results show that the vortex length is shortened in gas-liquid annular flow, compared with that in single-phase gas flow. The pressure recovery length is closely related with the vortex length, and shorter vortex length leads to shorter pressure recovery length. The gas-liquid distribution suggests that flow around the apex of back-cone is very stable, little liquid is entrained into the vortex, and no liquid appears around the low pressure tapping, which makes a more stable pressure at the apex of cone sensor feasible. This finding highlights the importance of obtaining the low pressure from the back-cone apex, which should be recommended in the multiphase flow measurement. Our results may help to guide the optimization of the cone sensor structure in the wet gas measurement.

## 1. Introduction

The gas-liquid annular flow exists extensively in multiphase flow, especially in the wet gas flow (including the wet steam and the wet natural gas), where the gas volume fraction (GVF) is more than 95% [1]. For example, in the wet natural gas industry, the annular flow is one of three important flow patterns (the other two flow patterns are stratified flow, and slug flow) according to the flow pattern map proposed by the Shell Corporation [2]. Consequently, the measurement of gas-liquid annular flow is of great significance. There are many methods available to measure the annular flow—among which, the differential pressure (DP) method is the most popular one. 

As a typical DP meter, the cone meter, has been paid increasingly attentions in recent years for its advantages including stable signals, high accuracy, low pressure loss, wide range ability, and short installation requirements [3,4,5,6]. Like the classical orifice plate and Venturi tube meter, the measurement performance of cone meter was primarily related with the gas and liquid flow rates, the operating pressure, the diameter ratio and so on [7,8,9,10,11]. Due to the effects of the liquid, measurement of wet gas by cone meter are smaller than that by the orifice plate and the Venturi tube meter, hence it is favorable to improve the measurement accuracy of gas flow rate [10,12]. More and more measurement models and cone meters have been adopted in the measurement of wet gas. Stewart et al. [7] developed a measurement correlation of cone meter with different diameter ratio based on the method proposed by Steven [13]. Steven et al. [6,14] developed a measurement model by modifying the de Leeuw model [2]. The data used by the model is obtained from a cone meter with the diameter ratio of 0.75 and the inlet pipe diameters are four inches and six inches. He et al. [11] proposed an online measurement model to simultaneously measure the gas flow rate and the liquid flow rate in wet gas using one cone throttle device. A wet gas flowmeter named Roxar was developed by Emerson based on the cone meter [15]. It was successfully used in the flow measurement of the real oil-gas well. Although many investigations on the wet gas measurement by the cone meter have been conducted in the last two decades, researchers mainly paid attention to the influences of the flow parameters and the cone diameter ratio on the differential pressure measurement, and then develop measurement models. In contrast, the characteristics of wet gas through the cone sensor and its influence mechanisms on the measurement of pressure are not fully understood. 

Studies [2,11] show that the cone meter has potentials to predict the gas and liquid flow rates by using appropriate mathematical analysis. When the relationship between the pressure loss and the liquid flow rate, or the liquid phase fraction, was obtained, then the gas and liquid flow rates can be measured online by combining the corrected measurement correlations. How to determine the pressure recovery length of a cone sensor and obtain the pressure loss are of great significance. At present, few studies have been done on the pressure recovery length of the cone meter. In our previous study [9,16], we have investigated the gas and liquid two-phase flow pass the cone sensor. It was found that pressure recovery length of the cone meter was shortened in gas and liquid two-phase flow, compared with that of in the dry gas flow. Nonetheless, the corresponding mechanisms affecting the pressure recovery length did not be revealed. Researchers [10,12] also reported that the cone meter has excellent performance in the measurement of multiphase flow—however, the reasons are still unclear. The development of an accuracy yet cost-effective measurement technology based on cone meter remains a challenge. 

The cone sensor is an essential primary element of the cone meter. It plays an important but not fully understood role in the measurement of pressure, especially when used to measure the wet gas flow. Here, we choose the gas-liquid annular flow that most common in the wet gas to investigate its characteristics when flowing through a horizontal pipe with inserted cone sensor. The experiment and numerical simulation on the gas-liquid annular flow through cone were conducted in this paper. The numerical simulation method was verified and validated by the experiment. The effects of cone sensor on the structures of the annular flow, including the feature of the vortex behind the cone sensor, and the pressure recovery characteristics downstream of the cone sensor were investigated. Then the gas and the liquid phase distributions and their effects on the measurement of the pressure, the differential pressure and the velocity in the cross-section were analyzed.

## 2. Experimental Setup and Methods

### 2.1. Cone Test Section

**Figure 1:**
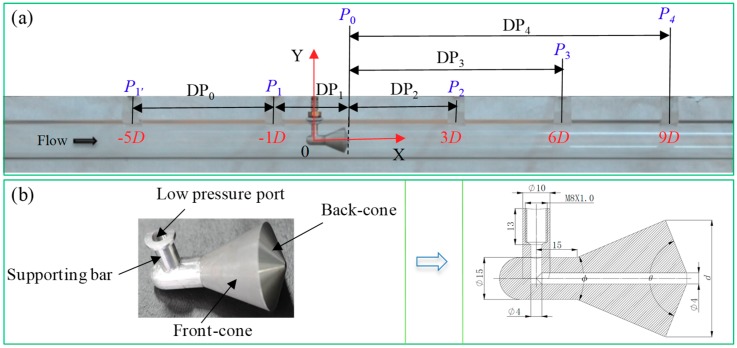
Test section (*β* = 0.55) (**a**) pressure tapping points distribution; (**b**) cone structure.

The test section and the cone sensor are shown in Figure 1. The cone sensor consists of two connected cones, i.e., a front-cone and a back-cone. The two cones are held by an ‘L’ shape supporting bar. The front- and back-cone angles (*φ* and *θ*) have been optimized with the respective cone angles are 45° and 135° (Figure 1b). The high pressure (e.g., *P*_1_) is obtained from pressure ports located upstream of cone sensor. Note that unlike the orifice plate and Venturi tube, the low pressure (e.g., *P*_0_) of the cone device passes through the cone, and is obtained from apex of the back-cone (Figure 1b). The pressure ports are located on the top of the test section as shown in Figure 1a.

Six pressures along the flow direction are measured. Among which two high pressure ports (*P*_1′_ and *P*_1_) locate at one times of the pipe diameter (1*D*) and five times of the pipe diameters (5*D*) upstream of the cone sensor, respectively. *P*_2_, *P*_3_, and *P*_4_ are three possible pressure recovery ports, which locate at 3*D*, 6*D* and 9*D* downstream of cone sensor, respectively. During the experiments, the *P*_4_, *DP*_0_, *DP*_1_, *DP*_2_, *DP*_3_, *DP*_4_ are measured directly, therefore the *P*_1′_, *P*_1_, *P*_0_, *P*_2_, and *P*_3_ can be obtained using the method shown in Table 1. 

**Figure 2:**
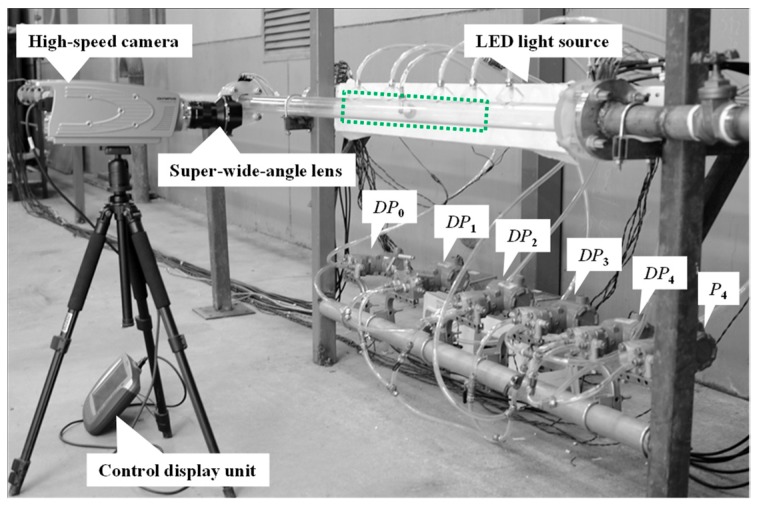
Photos of the measurement sensors.

The cone test section with the inlet inner diameter, *D*, of 50 mm is investigated. Figure 2 shows the DP sensors, the pressure sensors and the high-speed camera employed in the experiment. The transparent pressure transportation tubes are employed. During the experiment, we find that no liquid column accumulated in the transportation tubes. Therefore, there is little effect of liquid on the measurement of the static pressures. As displayed in Figure 2, the test section is horizontally positioned. To observe the flow structure of the gas-liquid annular flow, the plexiglass pipes are adopted. A high-speed camera whose model is *i*-SPEED TR, is used to record the flow structure. We also employ a “super wide angle lens” to capture the flow distributions from the upstream of the cone sensor to the downstream as large as possible. 

The equivalent diameter ratio, *β*, of the present cone test section is 0.55. Equation (1) provides the definition of *β*:

(1) $$\beta =\sqrt{\frac{D^{2}-d^{2}}{D^{2}}},$$

where *d* is the cone sensor throat diameter, as shown in Figure 1b.

### 2.2. Experimental Setup

**Figure 3:**
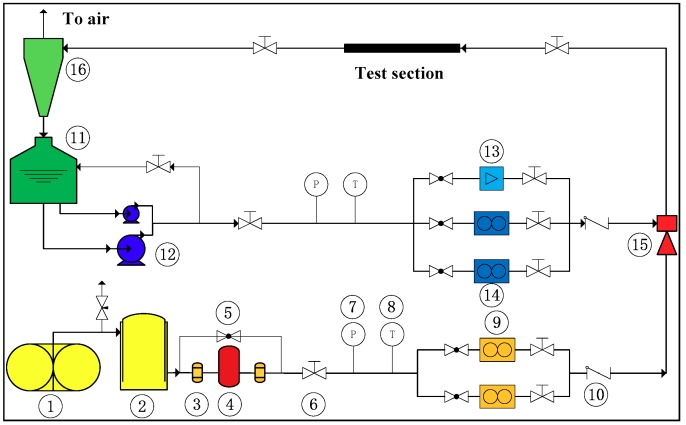
Flow diagram of flow loop: (1) Win-screw compressor; (2) air storage reservoir; (3) filter; (4) freezing dryer; (5) globe valve; (6) regulating valve; (7) pressure meter; (8) temperature gauge; (9) air Coriolis mass flow meter; (10) one-way valve; (11) water tank; (12) centrifugal pump; (13) electromagnetic flow meter; (14) water mass flow meter; (15) gas–liquid mixer; (16) gas–liquid separator.

The experiments were carried out in the Gas-Liquid Two Phase Flow Loop of Xi’an Jiaotong University. The flow diagram is shown in Figure 3. More information about the experimental apparatus are detailed in Ref. [5]. The measurement devices and the corresponding parameters are listed in Table 2. 

### 2.3. Experimental Method

The experiments focus on the effects of the gas and the liquid flow rates on the flow structure and the pressure distributions. Table 3 shows the experimental conditions. The *U_sg_* and *U_sl_*, are the superficial gas velocity and the superficial liquid velocity, respectively. They can be calculated using Equations (2) and (3). The operating pressure with approximately 0.2 MPa was tested, and the GVF of the test conditions is more than 98%.

(2) $$U_{sg}=\frac{4m_{g}}{\pi D^{2}\rho_{g}},$$

(3) $$U_{sl}=\frac{4m_{l}}{\pi D^{2}\rho_{l}},$$

where *m* and *ρ* are the mass flow rate and fluid density, respectively, *g* and *l* denote the gas and liquid, respectively.

The values and expanded uncertainty values of inlet pressure, temperature, gas mass flow rate, liquid flow rate, superficial gas velocity, superficial liquid velocity and gas volume fraction are shown in Table 3. The uncertainties are calculated according to the ISO/IEC GUIDE 98-3: 2008 [17]. Note that these uncertainties are calculated under the 95% confidence level.

## 3. Numerical Methods and Models

**Figure 4:**
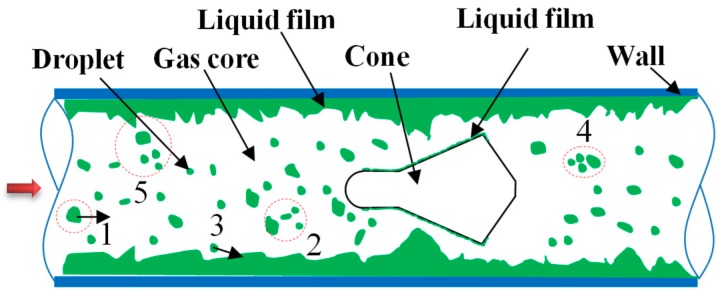
Interaction between gas and liquid when annular flow through pipe with inserted cone. 1—Original droplet; 2—Droplet breakup; 3—Impinging droplet; 4—Droplet coalescence; 5—Film separation and breakup.

A typical gas-liquid annular flow consists of gas core, droplet and liquid film, among which the gas and droplet flow in the central zone of the pipe, the liquid film is around the pipe wall. The dynamics phenomena such as the movement, separation and breakup of the liquid film, the entrainment and deposition of the film are generally occurred. The method to accurately capture the gas and liquid phase interface, to deal with interactions between the gas core and the liquid film and between droplets, are still a challenge in the multiphase flow simulation [18,19,20]. When the annular flow through the pipe with inserted cone sensor, near cone body, especially around the cone edge, the interactions among the droplet, the gas core, the liquid film and the cone body are more intense compared with that no inserted cone in the pipe (Figure 4). 

To accurately simulate the complex behaviors of annular flow through a pipe with inserted cone sensor, it is a huge expenditure of the computing resource and the computational convergence is difficult. Hence the following assumptions are made in the present simulation: (a) The annular flow is fully developed; the entrainment rate and deposition rate are in equilibrium. The droplets with the Sauter Mean Diameter (*d*_32_) are used to simulate the effects of droplet swarms on the flow; (b) ignore the interactions between the droplets and liquid film; (c) the transient processes are not considered. These assumptions have been validated by the numerical simulations of annular (-mist) flow in the Venturi tube [21,22,23]. The results showed that the assumptions were reasonable to simulate the steady characteristics of the flow through the Venturi tube. 

### 3.1. Geometry Model and Flow Domain

**Figure 5:**
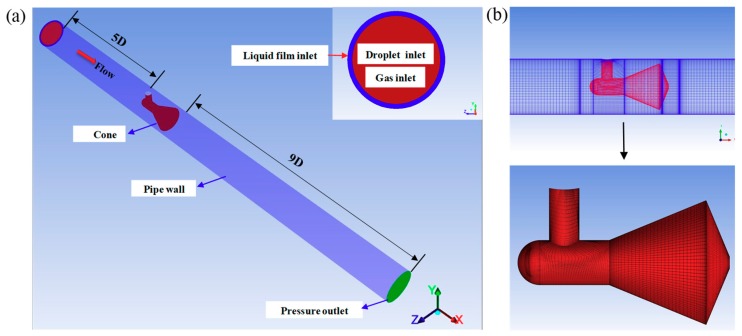
Geometry model and grid (*β* = 0.55) (**a**) geometry structure; (**b**) grid.

As shown in Figure 5a, the geometry structure of the simulated cone sensor is in accord with the test section shown in Figure 1. The upstream straight pipe length is 5*D* and downstream is 9*D*. The structured hexahedral meshes are used to mesh the flow domain (Figure 5b). The flow domain is meshed by the grid generation software “ANSYS ICEM CFD”. The grids are refined around the cone body for considering the high velocity and pressure gradient. The boundary layer grid is also added in the area close to the wall. As displayed in Figure 5a, the gas and droplet flow into the pipe from the circular region in the center of the pipe, and the liquid film from the annulus region. Owing to the liquid film thickness is not measured in present study, thus it is assumed to be uniform in circumference of pipeline [24].

### 3.2. Solving Strategies

In this work, the commercial software “ANSYS FLUENT” is employed for simulation and the steady simulations are conducted. Several typical multiphase flow models, i.e., the VOF (Volume of Fluid) model, the Mixture model, the Euler model and the DPM (Discrete Phase Model) model, are available in FLUENT. Among which the VOF model is used to track the shape and position of the free interface; while it consumes less computational resources and easier to converge than the Euler model. The DPM model can be used to calculate the trajectories of particles/droplets when the volume fraction of the discrete phase is less than 10% [25]. According to the gas-liquid annular flow through the cone sensor, the VOF coupled with the DPM model are employed in the present simulation. The interaction between the gas core and the liquid film is calculated by VOF model, and interaction between the gas core and the droplet is calculated by DPM model. The EWFM (Eulerian Wall Film Model) is adopted to calculate the shear effect between the gas and the liquid film. 

The liquid phase of the annular flow is consisting of the droplet and liquid film. The droplet volume fraction (entrainment fraction), the mass flow rate and the droplet size are several important parameters to describe the droplet. The entrainment fraction is critical for accurately simulating the flow characteristics. Hence an appropriate correlation to predict the entrainment fraction is very important. The entrainment fraction, *F_E_*, is calculated by Equations (4)–(7), which are obtained from the horizontal co-current gas-liquid two-phase annular flow [26]:

(4) $$F_{E}=F_{E,\max}\left(1-\exp\left(-We_{sg}/We_{sg}^{*}\right)\right),$$

(5) $$F_{E,\max}=F_{E,\max,\lim}\left[1-\exp\left(-{\left(\frac{Re_{sl}}{Re_{sl}^{*}}\right)}^{0.6}\right)\right],$$

(6) $$We_{sg}=\frac{\rho_{g}U_{sg}^{2}D}{\sigma }{\left(\frac{\rho_{l}-\rho_{g}}{\rho_{g}}\right)}^{1/4},$$

(7) $$Re_{sl}=\frac{\rho_{l}U_{sl}D}{\mu_{l}},$$

where $We_{sg}$ is the superficial gas Weber number, $We_{sg}^{*}$ is the analogous time constant, and is defined as the Weber number when entrainment fraction reaches 63.2% of its asymptotic value, *F_E_*_,max_ is the maximum entrainment fraction, *F_E_*_,max,lim_ is the limiting value of the maximum entrainment fraction, *Re_sl_* is the superficial liquid Reynolds number, $Re_{sl}^{*}$ is the analogous time constant in the form of a Reynolds number and is defined as the Reynolds number when maximum entrainment fraction reaches 63.2% of its limiting value, *σ* is the surface tension and *μ_l_* is the liquid viscosity.

The mass flow rate of droplet, *m_l,droplet_*, and the mass flow rate of liquid film, *m_l,film_*, in the inlet are calculated by Equations (8) and (9):

(8) $$m_{l,droplet}=m_{l}F_{E},$$

(9) $$m_{l,film}=m_{l}(1-F_{E}).$$

Thus the initial velocity of liquid film is obtained by Equations (10) and (11):

(10) $$U_{f}=m_{f}/\left(\rho_{l}A_{f}\right),$$

(11) $$A_{f}=\pi \left(D^{2}-{\left(D-2\delta \right)}^{2}\right)/4,$$

where *A_f_* is the area of annulus and *δ* is the thickness of the annulus.

The droplet size is another critical parameter affecting the simulation results. This has been reported by previous studies of the Venturi tube [27,28]. The Sauter Mean Diameter (*d*_32_) is employed here, which is calculated based on Equation (12) [29]. The *d*_32_ is corrected considering the effect of pipe diameter:

(12) $$d_{32}=c\left[0.37U_{sg}^{0.5}+0.602{\left(\frac{U_{sl}}{\rho_{g}U_{sg}}\right)}^{0.5}\right]\frac{\sigma }{\rho_{g}U_{sg}^{2}},$$

where *c* is the correction coefficient. 

In the DPM model, interaction of the droplets with continuous phase is considered. The numbers of continuous phase iterations per DPM iteration are 20. The Discrete Random Walk Model is also adopted for tracking the droplets. 

The Re-Normalization Group *k*-*ε* model (RNG *k*-*ε*) is used. The RNG *k-ε* model accounts for the effects of smaller scales of motion and improve the accuracy for rapidly strained flows and the swirling flows [25]. For the simulation of the flow through cone throttle device, the RNG *k-ε* model is found to be a very robust and accurate turbulence model [4,16]. The Scalable Wall Functions are employed, for which the y^+^ from 5–30 to 200–400 is recommended by the ANSYS FLUENT Theory Guide [25]. The constants in the RNG *k*-*ε* model are the standards values provided by Yakhot and Orszag [30]. The Dirichlet boundary condition is adopted at the inlet and the outlet, the velocity of the gas and liquid are set at the pipe inlet and the static pressure is set at the outlet (Figure 5). The turbulence intensity at the inlet and outlet is dependent on the empirical correlation for fully-developed duct flows. In the pressure-velocity coupling, the SIMPLEC scheme is used for quick convergence. For the spatial discretization, the pressure is calculated by PRESTO! the density, momentum, volume fraction, energy, turbulent kinetic energy and turbulent dissipation rate are calculated by QUICK. To improve the solution behavior of flow simulations when higher order spatial discretizations are used, the High Order Term Relaxation method is employed. The convergence criteria are assumed to be met when the iteration residuals are reduced to 10^−6^ and the mass flow rates of the inlet and outlet are equal. 

### 3.3. Mathematical Model

#### 3.3.1. Continuity Equation

The interface between the phases is tracked by the solution of one continuity equation for the volume fraction of one (or more) of the phases. For the steady incompressible flows, the continuity equation of the phase *q* can be expressed by Equation (13):

(13) $$\nabla \cdot \left(\alpha_{q}{\vec{v}}_{q}\right)=\frac{S_{\alpha_{q}}}{\rho_{q}},$$

where $\alpha_{q}$ is the volume fraction of phase *q*, ${\vec{v}}_{q}$ is the velocity of phase *q*, *ρ_q_* is the density of phase *q*, $S_{\alpha_{q}}$ is the source term, and is zero in the present simulation.

For the gas phase, the volume fraction equation will not be solved; and the gas phase volume fraction will be calculated by a constraint equation as shown in Equation (14):

(14) $$\alpha_{g}+\alpha_{l}=1,$$

where *α_g_* and *α_l_* is the gas volume fraction and the liquid volume fraction, respectively. 

The volume fraction equation is solved through the implicit time discretization. The fluid properties are obtained by taking a weighted average of the gas and liquid properties, on the basis of the gas and liquid volume fraction. The average density, *ρ*, and viscosity, *μ*, of gas-liquid mixture are calculated by Equations (15) and (16):

(15) $$\rho =\alpha_{g}\rho_{g}+\left(1-\alpha_{g}\right)\rho_{l},$$

(16) $$\mu =\alpha_{g}\mu_{g}+\left(1-\alpha_{g}\right)\mu_{l},$$

where *μ_g_* and *μ_l_* is the gas viscosity and liquid viscosity, respectively. 

#### 3.3.2. Momentum Equation

The momentum equation is connected with the volume fraction by the average density, *ρ*, and viscosity, *μ*, of gas-liquid mixture. One set of momentum equation for gas and liquid phase is solved, and the velocity fields are shared by two phases. The momentum equation is expressed by Equation (17):

(17) $$\nabla \cdot \left(\rho \vec{v}\vec{v}\right)=-\nabla P+\nabla \cdot \left(\bar{\bar{\tau }}\right)+\rho \vec{g}+F_{\mathrm{vol}},$$

(18) $$\bar{\bar{\tau }}=\mu \left(\nabla \vec{v}+\nabla {\vec{v}}^{T}\right)-\frac{2}{3}\nabla \cdot \vec{\nu }I,$$

where *P* is the static pressure, $\bar{\bar{\tau }}$ is the stress tensor, *I* is the unit tensor, *g* is the gravitational acceleration, *F*_vol_ is the external body forces that act on the gas and liquid interface, the surface tension is considered in present simulation (Equation (19)):

(19) $$F_{\mathrm{vol}}=\sigma \frac{\rho \kappa_{g}\nabla \alpha_{g}}{\frac{1}{2}\left(\rho_{g}+\rho_{l}\right)},$$

where $\kappa_{g}$ is the curvature. 

#### 3.3.3. Energy Equation

The temperature may change in the vicinity of cone sensor owing to the throttling effect of cone. Thus, the effect of temperature on the energy transfer is considered during the simulation. The energy equation is shared by both phases. The energy equation for the mixture is written as

(20) $$\nabla \cdot \left(\vec{v}\left(\rho E+P\right)\right)=\nabla \cdot \left(k_{eff}\nabla T\right),$$

where *k_eff_* is the effective conductivity, and shared by the mixture, *T* is the temperature and *E* is the energy. 

The VOF model treats the temperature, *T*, and the energy, *E*, as the mass-averaged variables:

(21) $$E=\frac{\sum_{q=1}^{n}\alpha_{q}\rho_{q}E_{q}}{\sum_{q=1}^{n}\alpha_{q}\rho_{q}},$$

where *E_q_* for each phase is based on the shared temperature and the specific heat of that phase.

#### 3.3.4. Turbulence Model

Equations (17) and (18) are closed by a turbulence model, i.e., the RNG *k*-*ε* model in the present simulation. This model is derived based on the “renormalization group” (RNG) methods [30]. Compared with the standard *k*-*ε* model, the turbulence kinetic energy (*k*) and its dissipation rate (*ε*) in the transport equations are modified. The transport equations of the RNG *k*-*ε* model are as follows:

(22) $$\rho u_{i}\frac{\partial k}{\partial x_{i}}=\frac{\partial }{\partial x_{j}}\left(\alpha_{k}\mu_{eff}\frac{\partial k}{\partial x_{j}}\right)+G_{k}+G_{b}-\rho \varepsilon ,$$

(23) $$\rho u_{i}\frac{\partial \varepsilon }{\partial x_{i}}=\frac{\partial }{\partial x_{j}}\left(\alpha_{\varepsilon }\mu_{eff}\frac{\partial \varepsilon }{\partial x_{j}}\right)+C_{1\varepsilon }\frac{\varepsilon }{k}G_{k}-C_{2\varepsilon }\frac{\rho \varepsilon^{2}}{k}-R_{\varepsilon },$$

where *μ_eff_* is the effective viscosity, *α_k_* and *α_ε_* are the inverse effective Prandtl numbers for *k* and *ε*, respectively, *α_k_* = *α_ε_* ≈ 1.393, *C*_1*ε*_ and *C*_2*ε*_ are constants, *C*_1*ε*_ = 1.42, *C*_2*ε*_ = 1.68. *G_k_* is the production of turbulence kinetic energy and is defined as

(24) $$G_{k}=\mu_{t}S^{2},$$

where *S* is the modulus of the mean rate-of-strain tensor and is calculated by Equations (25) and (26):

(25) $$S=\sqrt{2S_{ij}S_{ij}},$$

(26) $$S_{ij}=\frac{1}{2}\left(\frac{\partial u_{i}}{\partial x_{j}}+\frac{\partial u_{j}}{\partial x_{i}}\right),$$

*G_b_* in Equation (22) is the production of turbulence due to buoyancy as the gravity is considered. For idea gas, *G_b_* is given by

(27) $$G_{b}=-g_{i}\frac{\mu_{t}}{\rho {\mathrm{Pr}}_{t}}\frac{\partial \rho }{\partial x_{i}},$$

where *g_i_* is the component of the gravitational vector in the *i*th direction and Pr*_t_* is the turbulent Prandtl number for energy. 

The equations for turbulent viscosity are expressed as

(28) $$d\left(\frac{\rho^{2}k}{\sqrt{\varepsilon \mu }}\right)=1.72\frac{\overset{⌢}{\upsilon }}{\sqrt{{\overset{⌢}{\upsilon }}^{3}-1+C_{\upsilon }}}d\overset{⌢}{\upsilon },$$

(29) $$\overset{⌢}{\upsilon }=\frac{\mu_{eff\;}}{\mu }$$

where *C_υ_* is a constant, and *C_υ_* ≈ 100. 

In the high-Reynolds number limit, Equation (28) gives $\mu_{t}=\rho C_{\mu }k^{2}/\varepsilon$, in which *C_μ_* = 0.0845. 

*R_ε_ in* Equation (23) is the rapid strain term and is written as

(30) $$R_{\varepsilon }=\frac{C_{\mu }\rho \eta^{3}\left(1-\eta /\eta_{0}\right)}{1+\gamma \eta^{3}}\frac{\varepsilon^{2}}{k},$$

where η=Sk/ε, *η*_0_ = 4.38, *γ* = 0.012.

#### 3.3.5. Droplet Equations

The trajectory of a droplet is tracked by Lagrangian method in the DPM model. For the fully developed annular flow in the pipe, the gravitational acceleration, *g*, and the drag force, *F_D_*, between phases are main forces acting on the droplet. Because the velocity around the cone change largely, the effects of the Saffman’s lift force, *F_s_*, and pressure gradient force, *F_d_*, on the motion of droplet are also considered [31]. Equations of motion for droplet are written as Equations (31)–(34):

(31) $$0=F_{D}\left(u_{g}-u_{d}\right)+\frac{g\left(\rho_{d}-\rho_{g}\right)}{\rho_{d}}+F_{x},$$

(32) FD=18μgρddd2CDRed24,

(33) Red=ρgdd|ud−ug|μg,

(34) CD=a1+a2Red+a3Red2,

where *u_g_*, *u_d_* is the gas velocity and droplet velocity, respectively, *ρ_d_* is the droplet density, *d_d_* is the droplet diameter, *F_x_* is the additional force term, the Saffman’s lift force, *F_s_*, and pressure gradient force, *F_d_*, are included in present simulation, Re*_d_* is the relative Reynolds number, which is defined as Equation (33), *C_D_* is the drag coefficient, *a*_1_, *a*_2_, *a*_3_ are empirical constants which are obtained from the smooth spherical droplets. These constants are obtained over several ranges of droplet Reynolds number [32].

The Saffman’s lift force [33] caused by the shear is expressed by:

(35) $$F_{s}=\frac{2K_{s}\nu^{1/2}\rho d_{ij}}{\rho_{d}d_{d}{\left(d_{lk}d_{kl}\right)}^{1/4}}\left(u-u_{d}\right),$$

where *ν* is the kinematic viscosity, *d_ij_* is the deformation tensor, and *K*_s_ = 2.594.

The pressure gradient force in the fluid is expressed by:

(36) $$F_{d}=\frac{\rho }{\rho_{d}}u_{d}\frac{\partial u}{\partial x},$$

### 3.4. Model Verification and Validation

#### 3.4.1. Grid Convergence Verification

**Figure 6:**
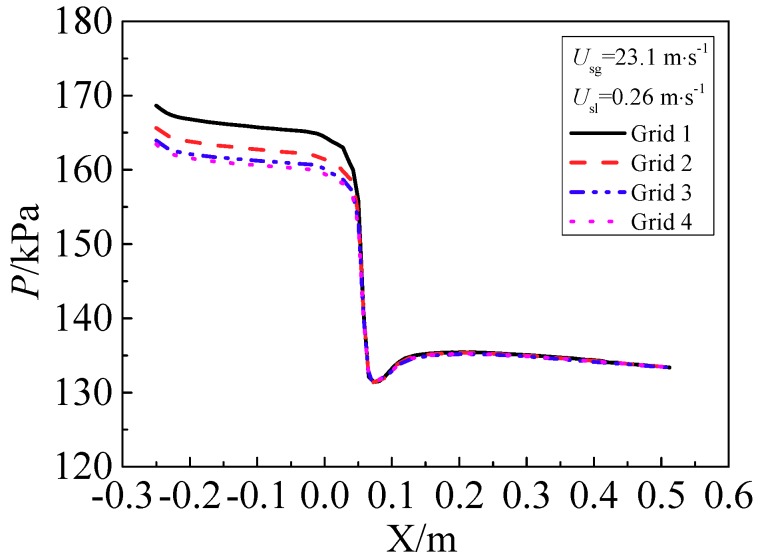
Wall pressure profiles under different grids.

Four groups of grids with different grid resolutions are used to verify the grid convergence (Table 4). The globe grid refinement method is employed, and the grid refinement ratio is approximate to 2 [34]. The y^+^ under different grid numbers is listed in Table 4. We can see that the y^+^ value is between the recommended values of the RNG *k*-*ε* model and the Scalable Wall Functions in ANSYS FLUENT. As shown in Figure 6, with the increase of the grid number, the static pressure of the wall predicted by Grid 4 changes little compared with the pressure predicted by Grid 3. Table 5 shows five typical pressures under different grid numbers. A parameter, *ε*, is adopted to investigate the changes of pressure with grid numbers. *ε* is defined as

(37) $$\varepsilon_{i+1,i}=\frac{P_{i+1}-P_{i}}{P_{i}}\times 100\%,$$

where *P_i_* and *P_i_*_+1_ is the pressure predicted by the coarse grid and the finer grid, respectively. 

It is found that the change of the pressure is less than 0.75% when the grid number increases from Grid 3 to Grid 4. Hence Grid 3 is sufficient for simulating the flow through the cone sensor. 

#### 3.4.2. Model Validation

**Figure 7:**
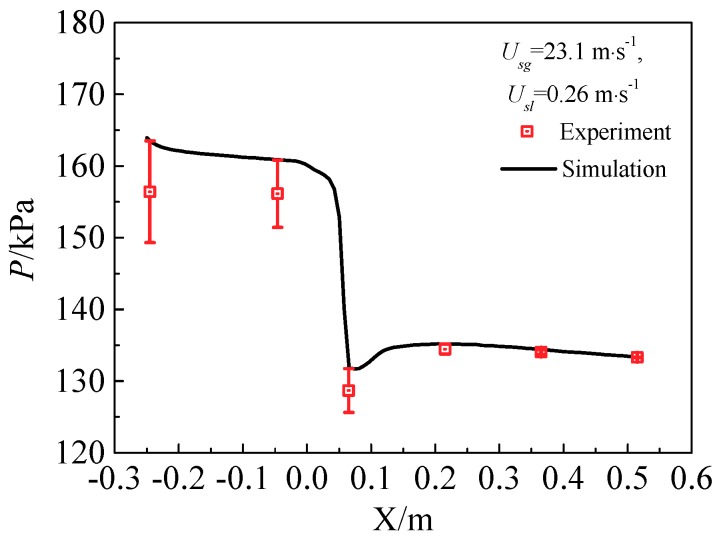
Comparison of simulated wall pressure profile with experimental results.

The wall pressure profile is displayed in Figure 7. Several typical pressures with uncertainties measured in the experiment and the values predicted by simulation are shown in Table 6. Comparison of the wall pressure profile between the numerical simulation results and the experiments shows that the predicted pressure agrees well with the experimental results, and the relative error is less than 5.0%. The results demonstrate that the numerical model and simulation method are reasonable and credible. 

## 4. Results and Discussion

### 4.1. Characteristics of Vortex and Its Effects on Pressure Recovery

#### 4.1.1. Vortex Downstream of Cone Sensor

**Figure 8:**
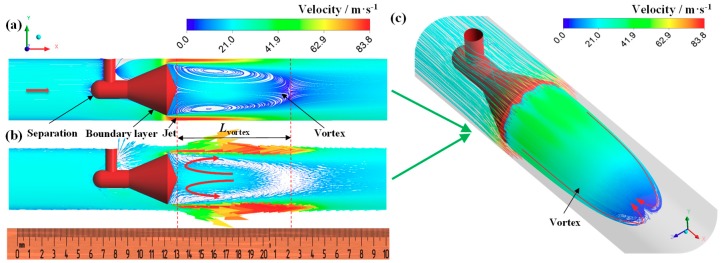
Streamline and velocity vector distribution under single-phase gas flow (*P* = 0.2 MPa, *U_sg_* = 21.3 m·s^−1^, *U_sl_* = 0 m·s^−1^, GVF = 100%). (**a**) Streamline; (**b**) velocity vector; (**c**) vortex behind cone.

Figure 8 shows the streamline and velocity vector as inlet flow is the single-phase gas flow. It is can be seen that the annular jet is formed around cone throat. The velocity reaches the maximum at certain position downstream of cone throat (Figure 8b). A large scale vortex is formed behind the cone owing to the shearing action of the jet, which leads the increase of the pressure gradient behind the cone. The velocity in the vortex center is low and reaches the minimum at the endpoint of vortex (the endpoint of vortex is the position where the velocity is approximately zero). The length of the vortex (*L*_vortex_) is two to three times of the diameter of the cone (*L*_vortex_ ≈ 2–3*d*). Flow direction of vortex near the pipe wall is as same as the main flow, and the flow direction of vortex in the center region of pipe is opposite to the main flow, as shown in Figure 8b,c. 

**Figure 9:**
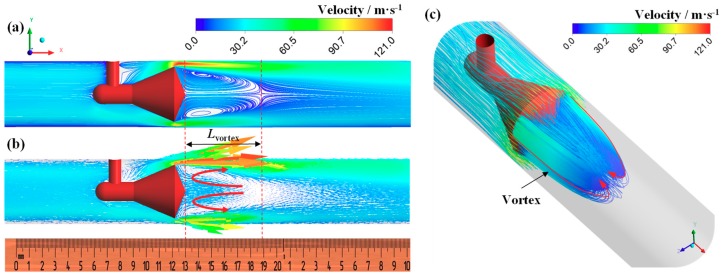
Streamline and velocity vector distribution under annular flow (*P* = 0.2 MPa, GVF = 98.53%, *U_sg_* = 21.1 m·s^−1^, *U_sl_* = 0.32 m·s^−1^). (**a**) Streamline; (**b**) velocity vector; (**c**) vortex behind cone.

As shown in Figure 9, the feature of vortex in the gas-liquid annular flow and the single-phase gas flow is similar, whereas the length of vortex in the annular flow is shorter. Take the cases in Figure 8 and Figure 9 for example, *L*_vortex_ in Figure 9 is reduced by 36% compared with that in Figure 8. The probable influence mechanisms are as follows. (a) Effects of fluid property: The mean density and viscosity of gas-liquid mixture are much higher than the single-phase gas. Thus, the Reynolds number of gas-liquid mixture is increased compared with the single-phase gas (the increase of the density is larger than the increase of the viscosity, and the velocity of the annular jet in the annular flow is much higher than that in the single-phase gas). Therefore, the momentum exchange between the air and the liquid is enhanced, the energy loss also increases. Hence the development of the vortex is suppressed. (b) Effects of throat jet: The gas-liquid annular jet impinges on the pipe wall, the liquid rebound and some breakup into droplets; at the same time, some liquid is entrained into the vortex, leading to the increases of energy loss of the vortex. Furthermore, we also find in the experiment that the rebound height of the liquid increases with the liquid flow rate increasing [9]. This also hinders the development of the vortex. 

**Figure 10:**
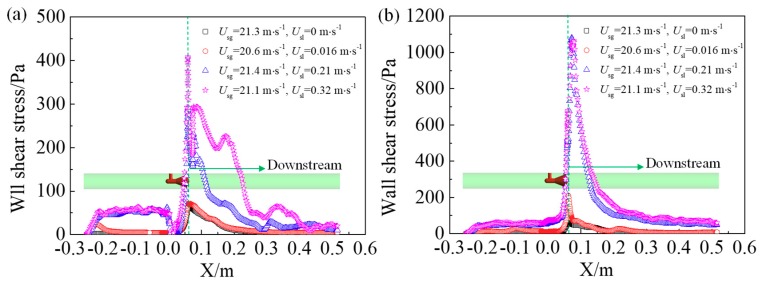
Wall shear stress at the top and bottom of the pipe (**a**) top; (**b**) bottom.

The wall shear stress distribution shown in Figure 10 can also be used to explain the variation of the vortex length in the gas-liquid annular flow and the single-phase gas flow. The shear stress at the top and bottom of the pipe wall is displayed here. Results show that the shear stress is higher in the gas-liquid two phase flow than that in the single phase flow, and it increases with the superficial liquid velocity. Therefore, the velocity gradient is higher in the gas-liquid two phase flow and the momentum exchange is enhanced. Furthermore, the shear stress direction and the flow direction of vortex near the wall is opposite, which hinders the movement of the vortex.

**Figure 11:**
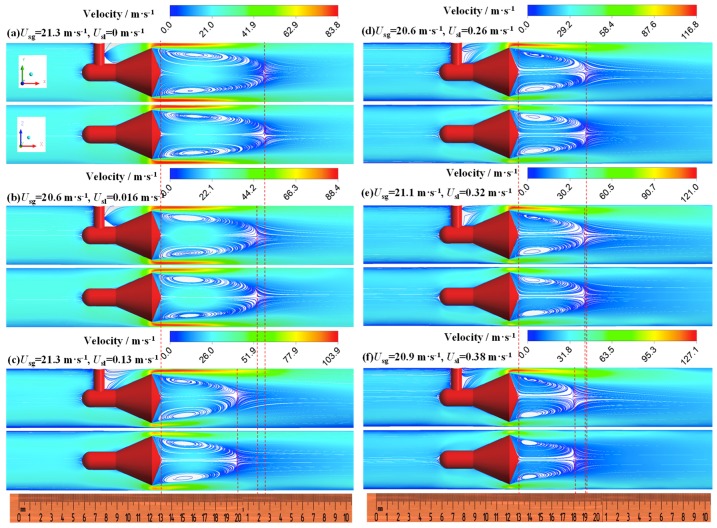
Effect of liquid velocity on vortex (**a**) GVF = 100%; (**b**) GVF = 99.93%; (**c**) GVF = 99.41%; (**d**) GVF = 98.77%; (**e**) GVF = 98.53%; (**f**) GVF = 98.23% (up: XOY cross section, down: XOZ cross section, ruler unit: mm).

**Figure 12:**
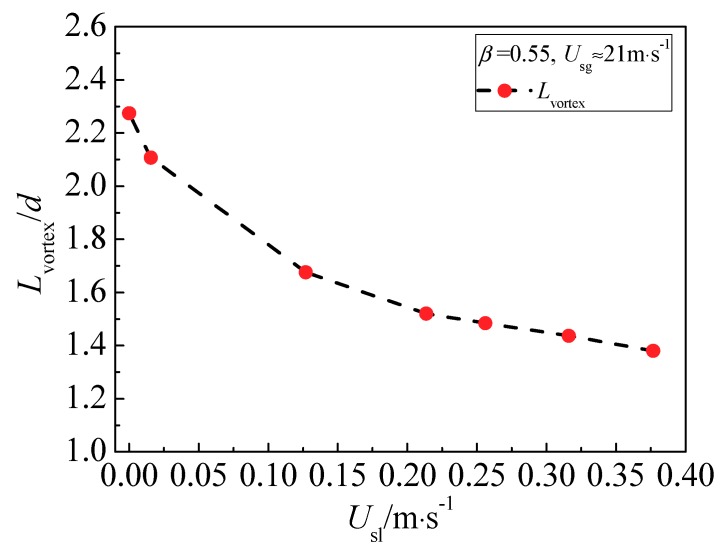
Relationship between vortex length and liquid velocity.

In the annular flow, the effects of superficial liquid velocity on the vortex behind the cone are shown in Figure 11. We find that the vortex is symmetrical along the axial under the single-phase gas and the gas-liquid annular flow. The symmetry of the vortex is virtually independent of the superficial liquid velocity, especially in the XOZ cross section. The length of the vortex (*L*_vortex_) reduces with the superficial liquid velocity increasing. Figure 12 shows that *L*_vortex_ is monotonically decreased with the superficial liquid velocity. In addition, *L*_vortex_ is increased as the superficial gas velocity increasing and almost not affected by the operating pressure.

#### 4.1.2. Effects of Vortex on Pressure Recovery Length

As we have stated in Introduction Section, the pressure recovery length is of great significance for developing an online measurement method to obtain the gas and liquid flow rates simultaneously based on one throttle sensor. According to Reader-Harris [35], the pressure recovery length of a DP meter is defined as the distance from the outlet of the throttle element to the location where the pressure is almost unchanged. Downstream of the pressure recovery location, the pressure is reduced as the results of the fluid frictions and the frictions between pipe wall and fluid. Therefore, the pressure recovery positions can be determined by comparing the static pressure at different positions downstream of cone sensor. Six pressure ports of the present cone test section are displayed in Figure 1. The pressure recovery positions of the cone meter can be obtained through the method as follows [9]: (a) When *P*_2_ > *P*_3_ > *P*_4_, i.e., *DP*_3-2_ = *P*_3_ − *P*_2_ < 0 and *DP*_4-3_ = *P*_4_ − *P*_3_ < 0, the pressure is recovered at 3*D* downstream of the cone sensor (*P*_2_); (b) when *P*_2_ < *P*_3_ > *P*_4_, i.e., *DP*_3-2_ > 0 and *DP*_4-3_ < 0, the pressure has not been recovered at 3*D*, while it can be recovered at 6*D* downstream (*P*_3_). 

**Figure 13:**
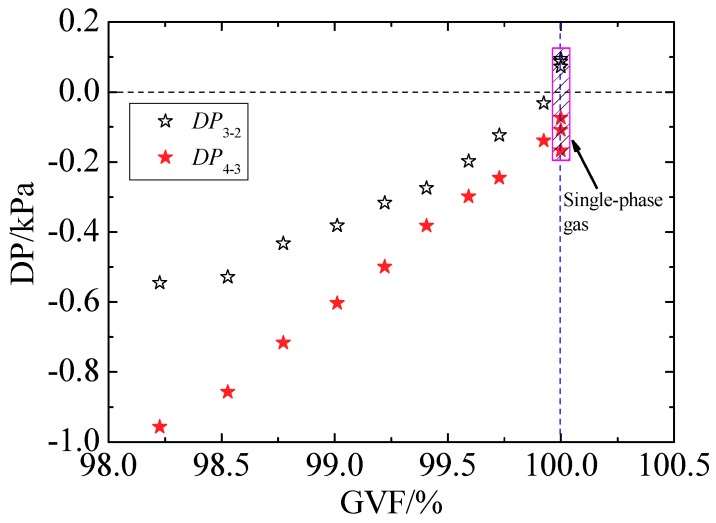
Pressure difference between pressure tapings downstream of cone.

As shown in Figure 13, when the inlet flow is single-phase gas (i.e., GVF = 100%), it can be seen that *DP*_3-2_ > 0 and *DP*_4-3_ < 0, so the pressure has recovered at 6*D* position (*P*_3_); if the inlet flow is gas-liquid annular flow (i.e., GVF < 100%), it is find that *DP*_3-2_ < 0 and *DP*_4-3_ < 0, so the pressure has recovered at 3*D* downstream (*P*_2_). The results demonstrate that the pressure recovery length is shortened in the gas-liquid annular flow compared with that in the single-phase gas flow. 

**Figure 14:**
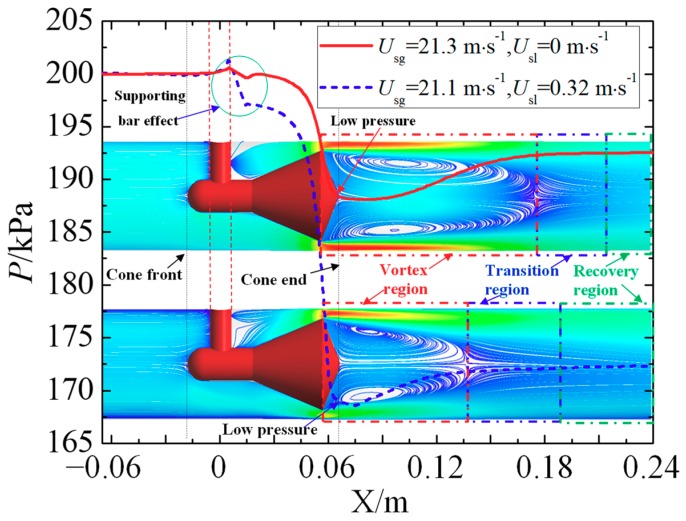
Effect of vortex on the wall pressure.

To gain a further understanding of the reason the pressure recovery length is shortened in the annular flow, the effect of vortex on the static pressure along the flow direction is shown in Figure 14. From the point of view of the static pressure, the flow downstream of the cone sensor is divided into three regions, i.e., the vortex region, the transition region and the recovery region. The static pressure decreases gradually flowing though the cone and reaches the minimum value at certain position of the vortex region, and then increases. After the transition region, when the streamline tends to flow parallel with the mainstream, the pressure is considered recovered. We conclude that the pressure recovery length is closely related with the vortex length. Longer vortex length needs longer pressure recovery length. As we have discussed above, the *L*_vortex_ in the annular flow has been shortened with respect to that in the single-phase gas. Hence much shorter pressure recovery length is needed for the annular flow. The results uncover a pathway that optimizes the structure of cone sensor to reduce the vortex length and thus reduce the pressure recovery length, which may help to develop a more compact cone meter. 

**Figure 15:**
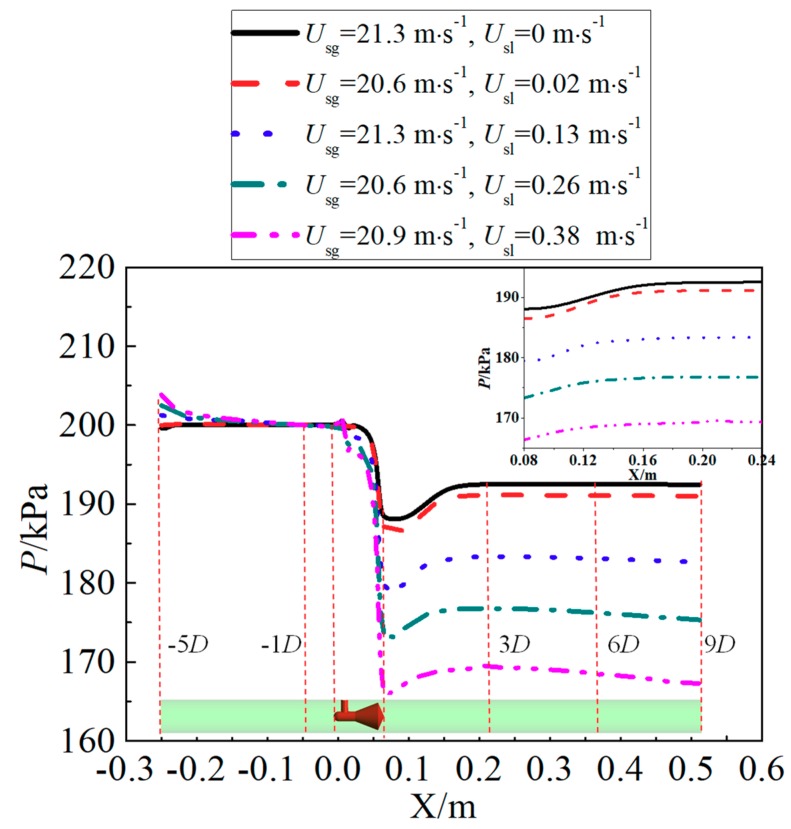
Wall static pressure along flow direction under different superficial liquid velocity.

Figure 15 shows that the differential pressure produced by the cone sensor increases as the superficial liquid velocity rises, while the pressure downstream of cone fells. The pressure will recover more quickly when the liquid velocity is increased, hence shorter pressure recovery length is required.

### 4.2. Gas-Liquid Distribution

**Figure 16:**
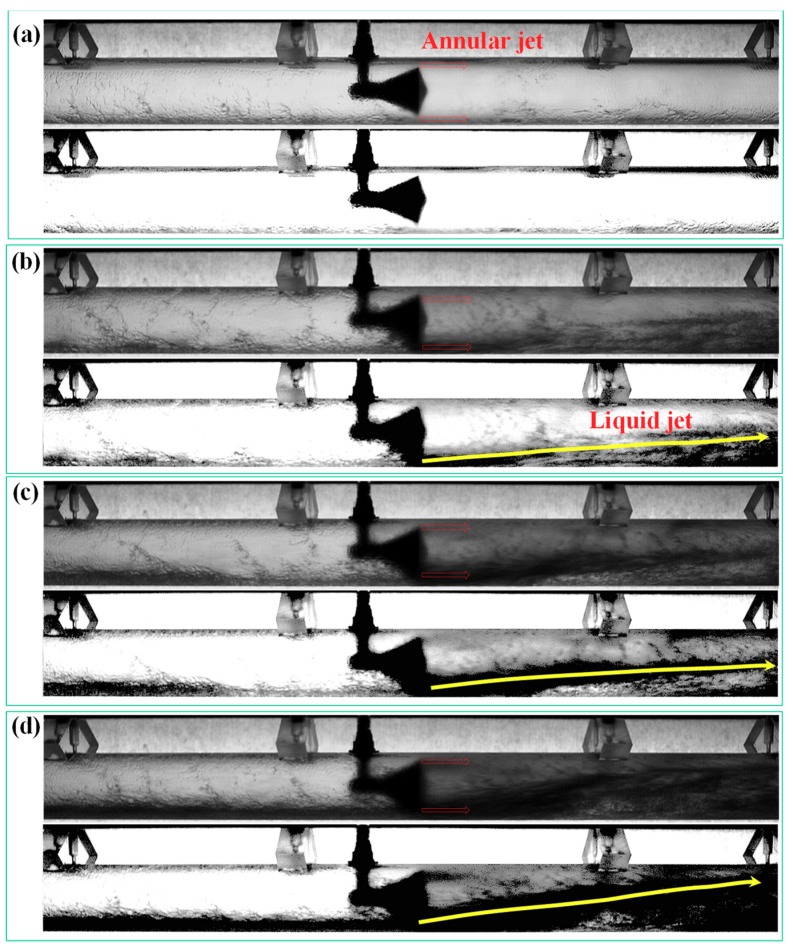
Variation of flow pattern under different superficial liquid velocity (**a**) U*_sg_* = 20.6 m·s^−^^1^, U*_sl_* = 0.016 m·s^−^^1^, GVF = 99.93%; (**b**) U*_sg_* = 21.3 m·s^−^^1^, U*_sl_* = 0.13 m·s^−^^1^, GVF = 99.41%; (**c**) U*_sg_* = 20.6 m·s^−^^1^, U*_sl_* = 0.26 m·s^−^^1^, GVF = 98.77%; (**d**) U*_sg_* = 20.9 m·s^−^^1^, U*_sl_* = 0.38 m·s^−^^1^, GVF = 98.23% (up: Enhanced image, down: Binary image, the videos can be found in Supplementary Materials).

In the horizontal annular flow, the liquid film is asymmetrically distributed around the pipe wall because of the effect of gravity. Generally, the liquid film at the top of the pipe is thinner than the film at the bottom (Figure 16). When superficial liquid velocity is low, as shown in Figure 16a, the cone sensor has little influence on the flow pattern. As the superficial liquid velocity increasing, the liquid film thickness will increase correspondingly. When the film thickness is larger than the width of the annular channel, the liquid jet is formed, as shown in Figure 16b–d. In the downstream of the cone sensor, some liquid is entrained into the gas core and mixed with the gas. The corresponding videos are available in Supplementary Materials: Videos S1–S4.

**Figure 17:**
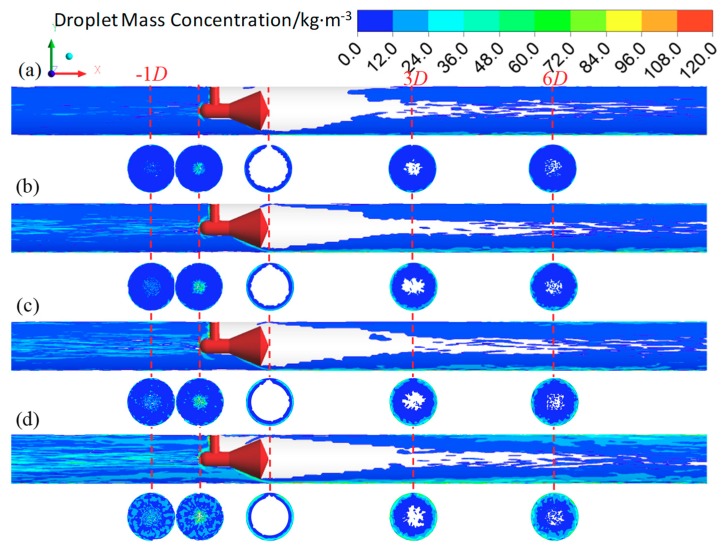
Droplet mass concentration under different liquid velocity. (**a**) *U_sg_* = 21.3 m·s^−1^, *U_sl_* = 0.13 m·s^−1^; (**b**) *U_sg_* = 21.4 m·s^−1^, *U_sl_* = 0.21 m·s^−1^; (**c**) *U_sg_* = 20.6 m·s^−1^, *U_sl_* = 0.26 m·s^−1^; (**d**) *U_sg_* = 20.9 m·s^−1^, *U_sl_* = 0.38 m·s^−1^.

In the present simulation, interactions between the gas core and droplet are calculated by DPM model. When the discrete droplets flow past the cone, the annular droplet flow is formed downstream of the cone (Figure 17). This is affected by the annular throttle channel and the gas-liquid jet with high velocity (e.g., the jet velocity in Figure 16d is up to 127 m·s^−1^). Note that there are some differences in gas-liquid distribution between the simulation and the experiment. This is primarily caused by the difference of the liquid film in the inlet, which is assumed to be uniform and symmetrical in circumference of pipeline during the simulation. If the liquid film thickness is measured and the interaction with the droplets is calculated, it is possible to simulate the liquid jet in Figure 16. 

**Figure 18:**
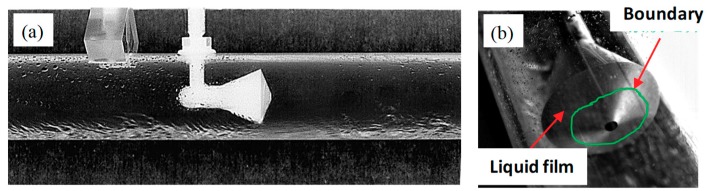
Liquid distribution on cone observed in experiment (**a**) front view of flow through cone sensor; (**b**) liquid film around the low pressure tapping (the video is available in Supplementary Materials: Video S5).

Figure 17 also shows that little liquid is entrained into the vortex region. The droplet in the gas core of the annular flow will increase along the flow direction. We also find that some liquid will accumulate on the front-cone surface, which is observed in the experiment (Figure 18) and consistent with our previous study [16]. As shown in Figure 17, the back-cone is covered with a liquid film, whereas there is a film “boundary” around the low pressure tapping where no liquid appears around it. The video is available in Supplementary Materials: Video S5. The liquid distribution around the back-cone apex suggests that a “dry surface” exists around the low pressure tapping, which makes it in a stable condition. This is beneficial to measure the low pressure. 

**Figure 19:**
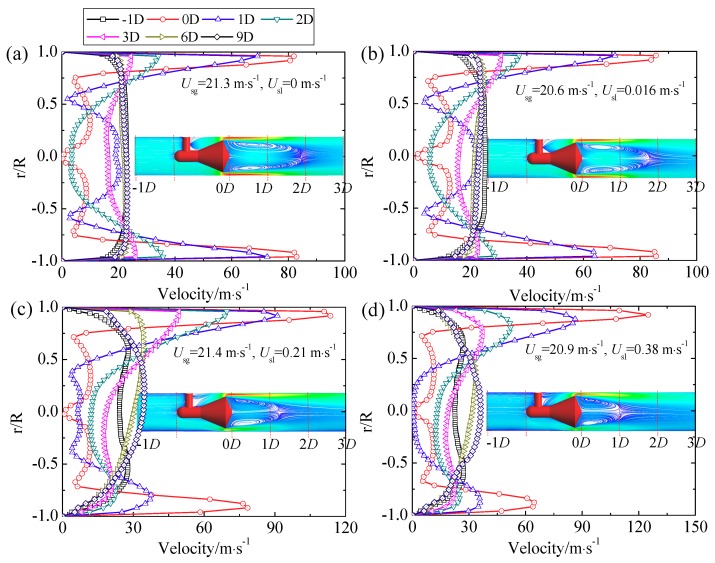
Velocity along Y axis direction under different cross sections. (**a**) *U_sg_* = 21.3 m·s^−1^, *U_sl_* = 0 m·s^−1^; (**b**) *U_sg_* = 20.6 m·s^−1^, *U_sl_* = 0.016 m·s^−1^; (**c**) *U_sg_* = 21.4 m·s^−1^, *U_sl_* = 0.21 m·s^−1^; (**d**) *U_sg_* = 20.9 m·s^−1^, *U_sl_* = 0.38 m·s^−1^.

The velocity is also closely related with the gas-liquid distribution and the vortex downstream of the cone sensor. The velocity along the Y axis in the cross section upstream and downstream of the cone sensor is shown in Figure 19. The 0*D* is the cross section at the apex of the back-cone, which is also the cross section of the low pressure tapping. It is found that the velocity around the apex of back-cone (*r*/*R* = 0, 0*D* cross section) is approximately zero both in the single-phase flow and the annular flow. This demonstrates that the flow around the apex of back-cone is very stable, which is beneficial to the measurement of low pressure. As the liquid velocity increase, the maximum velocity at the bottom of the pipe is lower than the velocity at the top. This is because the liquid film at the bottom is thicker than at the top. The velocity in the cross section out of the vortex region, such as the 3*D* cross section, is also affected by the gas and liquid distribution of the annular flow. 

The above analysis on the flow structure and the velocity through the cone sensor provides evidences that the method to obtain the low pressure from the back-cone apex is favorable. This type of pressure measurement method should be recommended in the multiphase flow measurement, such as the measurement of gas-liquid annular flow.

## 5. Conclusions

Investigations on the gas-liquid annular flow through a horizontal cone sensor were conducted in this paper. The influences of the cone sensor on the structure of the gas-liquid flow were analyzed. The pressure recovery characteristics downstream of the cone sensor were explored, along with the gas-liquid distribution and its effects on the measurement of pressure and velocity, which were also analyzed. The results indicate that the vortex is formed behind the cone sensor. The vortex length in the gas-liquid annular flow, in contrast with the single-phase gas flow, is shortened. It is resulted from effects of the fluid property and the jet in the cone throat, which increase the energy loss and suppress the development of the vortex. The pressure recovery length is closely related with the vortex length, longer vortex length leads to longer pressure recovery length. The vortex length and the pressure recovery length are decreased with the superficial liquid velocity in the annular flow. Little liquid is entrained into the vortex, and no liquid appeared around the low pressure tapping. Our results also find that the flow around the apex of back-cone is very stable. All these findings are beneficial to the measurement of the low pressure, as they highlights the importance of obtaining the low pressure from the back-cone apex, and emphasizes the needs to recommend it in the multiphase flow measurement, such as the measurement of gas-liquid annular flow.

## Figures and Tables

**Table 1:** Calculations of pressures at different pressure ports.

Differential Pressure, *DP*/Pa	Operating Pressure, *P*/Pa
*DP*_0_ = *P*_1′_ − *P*_1_	*P*_1′_ = *DP*_0_ *+ P*_4_ − *DP*_4_ + *DP*_1_
*DP*_1_ = *P*_1_ − *P*_0_	*P*_1_ = *P*_4_ − *DP*_4_ + *DP*_1_
*DP*_2_ = *P*_2_ − *P*_0_	*P*_0_ = *P*_4_ − *DP*_4_
*DP*_3_ = *P*_3_ − *P*_0_	*P*_2_ = *DP*_2_ + *P*_4_ − *DP*_4_
*DP*_4_ = *P*_4_ − *P*_0_	*P*_3_ = *DP*_3_ + *P*_4_ − *DP*_4_

**Table 2:** Measurement devices employed in this article.

Device	Measurement Range	Uncertainty	Manufacturer
Air Coriolis mass flowmeter	0–700 kg·h^−1^	±0.5%	Siemens, Munich, Germany
Electromagnetic flowmeter	0.0076~0.76 m^3^·h^−1^	±0.2%	Yokogawa Electric, Tokyo, Japan
Water Coriolis mass flowmeter	0–10,000 kg·h^−1^	±0.1%	Siemens, Munich, Germany
Temperature sensor	0–60 °C	±0.15 °C	Xi’an Instruments Factory, Xi’an, China
Pressure sensor	0–1.0 MPa	±0.075%	Emerson Process Management, St. Louis, MO, USA
Differential pressure sensor	0–6.22 kPa 0–16.25 kPa 0–62.5 kPa	±0.075%	Emerson Process Management, St. Louis, MO, USA
High-speed camera	0–10,000 fps	-	Olympus, Southend-on-Sea, UK
Data acquisition board	48 input channels 80 kS·s^−1^	16 bits	National Instrumentation, Austin, TX, USA

**Table 3:** Test conditions and uncertainties of the measurement parameters.

Pressure, *P*_1*′*_/MPa	Temperature, *T/*°C	Gas Mass Flow Rate, *m_g_/*kg·h^−1^	Liquid Mass Flow Rate, *m_l_/*kg·h^−1^	Superficial Gas Velocity, *U_sg_*/m·s^−1^	Superficial Liquid Velocity, *U_sl_*/m·s^−1^	Gas Volume Fraction, GVF/%
0.208 ± 0.000893	27.95 ± 0.17	537.13 ± 5.63	0	21.25 ± 0.27	0	100.00 ± 1.29
0.208 ± 0.000959	17.55 ± 0.17	540.13 ± 5.65	109.83 ± 0.66	20.62 ± 0.31	0.016 ± 0.000095	99.93 ± 1.62
0.206 ± 0.000918	20.65 ± 0.17	536.94 ± 5.63	400.88 ± 2.39	20.83 ± 0.29	0.057 ± 0.00034	99.73 ± 1.53
0.205 ± 0.000907	21.80 ± 0.17	554.73 ± 5.76	625.21 ± 3.73	21.65 ± 0.30	0.089 ± 0.00053	99.59 ± 1.50
0.206 ± 0.000909	22.18 ± 0.17	546.09 ± 5.69	896.84 ± 20.90	21.27 ± 0.29	0.13 ± 0.0030	99.41 ± 2.69
0.206 ± 0.000951	22.64 ± 0.17	547.75 ± 5.71	1185.17 ± 27.47	21.38 ± 0.29	0.17 ± 0.0039	99.22 ± 2.67
0.204 ± 0.000912	22.87 ± 0.17	542.96 ± 5.67	1507.74 ± 34.82	21.38 ± 0.29	0.21 ± 0.0049	99.01 ± 2.66
0.206 ± 0.000905	23.03 ± 0.17	526.54 ± 5.54	1808.50 ± 41.70	20.62 ± 0.28	0.26 ± 0.0060	98.77 ± 2.65
0.205 ± 0.000904	23.18 ± 0.17	537.86 ± 5.63	2231.62 ± 51.37	21.14 ± 0.29	0.32 ± 0.0074	98.53 ± 2.63
0.205 ± 0.000910	23.27 ± 0.17	531.49 ± 5.58	2659.96 ± 61.18	20.86 ± 0.28	0.38 ± 0.0087	98.23 ± 2.63

**Table 4:** Details of different meshes.

Grid	Number of Nodes	Number of Cells	y^+^
Grid 1	419,872	389,888	48–366
Grid 2	731,869	708,112	35–289
Grid 3	1,487,981	1,452,880	31–193
Grid 4	3,029,885	2,970,880	29–168

**Table 5:** Wall pressure and its changes with different grids.

Grid		Grid 1	Grid 2	Grid 3	Grid 4	*ε*_21_/%	*ε*_32_/%	*ε*_43_/%
Pressure/kPa	*P*_1′_	168,300.7	165,300.7	163,531.5	163,033.9	−1.78	−1.07	−0.3
	*P*_1_	164,510.3	162,310.3	160,835.9	160,241.3	−1.34	−0.91	−0.37
	*P*_0_	132,624.2	132,524.2	131,768.3	132,747.0	−0.075	−0.57	0.74
	*P*_2_	135,412.9	135,312.9	135,176.2	135,210.3	−0.074	−0.1	0.025
	*P*_3_	134,625.0	134,525.0	134,376.3	134,448.3	−0.074	−0.11	0.054
	*P*_4_	133,378.1	133,378.3	133,350.7	133,379.1	0.0001	−0.02	0.021

**Table 6:** Pressure uncertainty of the validation experimental data.

Pressure	*P*_1′_	*P*_1_	*P*_0_	*P*_2_	*P*_3_	*P*_4_
Experiment/kPa	156.42 ± 0.68	156.14 ± 0.64	128.70 ± 0.52	134.47 ± 0.55	134.06 ± 0.55	133.33 ± 0.52
Simulation/kPa	163.53	160.84	131.77	135.18	134.38	133.35
Relative error/%	4.55	3.01	2.38	0.53	0.24	0.016

## Supplementary Materials

The following are available online at http://www.mdpi.com/1424-8220/18/9/2923/s1, Video S1: U*_sg_* = 20.6 m·s^−^^1^, U*_sl_* = 0.016 m·s^−^^1^. Video S2: U*_sg_* = 21.3 m·s^−^^1^, U*_sl_* = 0.13 m·s^−^^1^. Video S3: U*_sg_* = 20.6 m·s^−^^1^, U*_sl_* = 0.26 m·s^−^^1^. Video S4: U*_sg_* = 20.9 m·s^−^^1^, U*_sl_* = 0.38 m·s^−^^1^. Video S5: Liquid distribution on cone surface.
